# Notices, Reviews, &c.

**Published:** 1859-07

**Authors:** 


### NOTICES, REVIEWS, &c.

*Medical and Literary Weekly*, $2 a year, Atlanta, Georgia, edited by Taliaferro & Thomas, we heartily commend to public patronage. It is very much like the Journal of Health in its objects, and is conducted with judgment and ability.

"*A Bachelor's Story*," by Oliver Bunce, $1, New York. published by the enterprising house of Rudd & Carlton, the mechanical getting up of whose publications is worthy of praise. The book itself abounds in lessons useful and true ; its sentiments are sound and practical, and can be read and re-read with interest and profit.

*Blackwood's Magazine* and the four Quarterlies, re-published for ten dollars a year, by Leonard Scott & Co., New York, afford to educated minds a large amount of most valuable reading for one year, at a very small cost.

*Scalpel Quarterly*, $1 a year, by Edward Dixon, M.D., is the most racy and instructive periodical of the times, as to the health, and manners, and habits of the people.

*The Fireside Monthly*, $1 50 a year, edited by W. W. Hall, 42 Irving Place, New York, is devoted to *Science, Literature, Art, and practical life* ; it is original, excludes fiction, and although not professedly a religious publication, is never, by any possibility, against evangelical religion. There is no similar publication in the world, and in the language of the Religious Herald of Richmond, Virginia, it is "a really useful work, deserving a place in every household." Are there enough religious, solid, conservative and reflecting men in the land, to support from principle, an experiment for supplying a monthly periodical for their families, which excludes all fiction, and deals in practical facts ? We shall see.

*Challen's Monthly*, $1 a year, is one of the cheapest and most instructive publications for christian families, among our exchanges.

*Merry's Museum*, $1 a year, New York, continues to be as useful as it is popular, among its multitudes of child-readers.

*Mother's Magazine*, New York, $1 a year, is worthy of an extensive patronage.

We see with pleasure, frequent quotations from the "*Happy Home*," Boston, $2 a year.

*The Home Monthly*, Buffalo, $1 50 a year, is creditable to the judgment and ability of its lady editors, Mrs. Arey and Gildersleeve.

*Godey's Lady's Book*, $3 a year, Philadelphia, has not in its twenty eight years' history, given a more interesting frontispiece than that for the July number, "Home and the Homeless," while—

*The Ladies' Home Magazine*, so long enriched by Arthur's pen, is as usual, always safe for family reading, and always instructive.

*Boston Medical and Surgical Journal*, $3 a year, is the veteran of New England medicals.

